# Sharpening the tip of the spear: Tailoring performance psychology for Special Operation Forces

**DOI:** 10.3389/fpsyg.2022.1068896

**Published:** 2022-12-09

**Authors:** Christian Ytterbøl, Dave Collins, Alan MacPherson

**Affiliations:** ^1^Norwegian Defence University College/Military Academy and UoE/Moray House School of Education and Sport (MHSES), Norwegian Defence University College, Oslo, Norway; ^2^UoE/Moray House School of Education and Sport (MHSES), University of Edinburgh, Edinburgh, Scotland, United Kingdom; ^3^UoE/Institute for Sport, Physical Education and Health Sciences (ISPEHS), University of Edinburgh, Edinburgh, Scotland, United Kingdom

**Keywords:** military, elites, combat, mental skills, performance

## Abstract

As performance psychology expands to address different environments, military settings are viewed as a natural extension. In certain cases, however, we suggest that a sub-optimal approach has been employed, due to a lack of specific knowledge of military culture and context, coupled with a diminished emphasis on conducting psychological research targeted directly on military performance. In this paper we explore the specific and importantly unique challenges encountered when researching and consulting with Special Operation Forces (SOF) within the North Atlantic Treaty Organization (NATO) alliance. To support both researchers and practitioners, we offer an overview of the current state of knowledge in this specific domain. We highlight key differences between SOF and conventional forces, then look at the specific requirements for developing performance psychology in the SOF context. Finally, we offer some perspectives on where opportunities might have been missed and offer some suggestions for more impactful (and accurate) research and practice.

## Introduction

For the North Atlantic Treaty Organization (NATO) members, the mantra of continually seeking to improve its personnel is a long-held consideration. This has led to the import and application of bodies of academic and practical knowledge sourced from pertinent performance domains, most notably sport (Rodden-Aubut and Tracey, 2022). Highlighting how soldiering has evolved, terms such as “soldier athletes,” (regarding the soldier as an athlete) and “tactical athletes,” (cit. LeDuc, 2018), are now used widely, without, we would argue, addressing the specificity that each subgroup possesses. For example, fighter pilots, infantry and SOF all are a part of the military, but their jobs are very different. Clearly, optimum service, research and understanding must cater for such in exactitudes (*cf.* Goodwin, 2008).

Using the right terminology is important as it clearly indicates the similarities and differences that practitioners and researchers should be aware of when working with SOF, as contrasted with General Purpose Forces (GPF). Importantly, psychologists looking to apply concepts developed in the sporting arena, or for GPF, need to be aware of the extreme differences of client and context involved. For example, most research conducted in military settings does not distinguish between differences in tactical military personnel, their roles, levels of training or expertise (Raabe et al., 2021). In other words, the special characteristics of SOF may go largely unnoticed or unaddressed.

Notably, this contention is not without precedent as papers addressing the “special natures” of specific populations have already been produced in domains such as music (Pecen et al., 2016) and dance (Henley, 2016). In addition, as we will suggest in this paper, certain methods have been either incorrectly applied or assumptions have been made that potentially limit the efficacy of the interventions proposed. Indeed, the increased focus on both physical and mental performance worldwide within tactical populations (e.g., police, military and firefighters) has created a more open forum for the implementation and delivery of psychological services. Consequently, it is both timely and important to evaluate current thinking and practice to engender critical debate and consider emerging avenues of enquiry.

Notably, however, peer-reviewed publications relating to psychology with tactical populations (i.e., encompassing emergency services and military) are sparse. In a recent systematic review of mental qualities and techniques in tactical populations, only 49 articles out of 7,220 potentially relevant publications met the inclusion criteria and specifically addressed the stated target (Raabe et al., 2021). In this paper specifically, the military population was regarded as one group, and distinct research focused on SOF was not mentioned. Based on our experience we would suggest that the special nature of SOF missions means that, as a group, they need specific research and implementation. In short, while the need for SOF theory underpinned by high-quality research is clearly stated and understood (Spencer, 2017) as yet, with the SOF community as its primary focus, the field of performance psychology requires further development.

Consequently, in this paper we firstly provide a brief overview of the current state of knowledge on SOF. We do this with reference to two key factors: namely, the term “special” and what that encompass. Secondly, we explore and characterize the term “performance” in the domain of SOF. Building upon these distinctions, we next provide a deeper understanding of the *demands of the arena* where these performances take place, especially as this influences implications for practitioner inquiry. Finally, we offer perspective as to where opportunities may have been overlooked. We start by taking an explicit look at the labels and nomenclature currently employed in this exacting field.

### Part 1: What are Special Operation Forces and what do they need?

Special Operation Forces (SOF) is comprised of operators who undergo rigorous selection and highly advanced training, with equipment tailored to the task at hand. Special operations were succinctly defined as follows: “Special operations encompass the use of small units in direct or indirect military actions focused on strategic or operational objectives. These actions require units with combinations of specialized personnel, equipment, and tactics that exceed the routine capabilities of conventional military forces” (Joint Special Operations University (U.S.) et al., 2015, p. 1). Therefore, SOF can be deployed across the spectrum of military operations, often with very little notice given and, at times, little specific preparation. As such, they are “specialist generalists,” retained in high demand by policy makers and with significant value placed upon their adaptive expertise (*cf.* Mees et al., 2020).

Furthermore, Searle (2017) suggests that SOF address tasks that are beyond the remit of operations undertaken by the regular military. The term “operations,” as used here, is defined broadly to include actions, activities, tasks, and missions. Importantly, however, we would take issue with this overly dichotomic conceptualization. In contrast, we posit that conventional forces and SOF are best thought of as distinct, *but* with a degree of overlap. Conventional operations are considered “inside the box”; conducting a standardized set of tasks (Searle, 2017, p. 7). Importantly, however, this does not accurately reflect the nature of recent military campaigns such as the global war on terror (GWOT), which demonstrates that “outside the box” tasks, such as Counter Insurgency operations (COIN) and some forms of Military Assistance (MA), can also be executed by conventional forces. In short, a “special operation” or at least a non-normal one, does not necessarily equate to the use of SOF! Importantly, however, SOF also go well beyond such a “special or routine” dichotomy, being designed to operate on an even wider spectrum of operations (Searle, 2017). Once again, their specialist-generalist characteristic is central to their role.

### SOF leadership

Another of several major differences is in the leadership structures used in GPF and SOF. For example, UK SOF leadership and organizational structures do not mirror the regular military. Officers commonly spend a comparatively short period of time with SOF, whereas enlisted “troopers,” dependent on their performance, can be with the squadron for up to 20 years. This has significant implications for how missions are conceived, planned, led, and reviewed. Membership of the troop is based upon expertise which may or may not be required to come to the fore at any given point during the lifecycle of an operation. Therefore, to operate effectively, there will be occasions when officers must follow experts of lower rank. In parallel, SOF operators must be leading where the context matches their expertise. This difference suggests that a “special” consideration of leadership is also needed.

As one example, consider the popular theory of Transformational Leadership (TL). This has been extensively researched in the context of regular military (Bass et al., 2003). TL posits that group performance can be linked to leaders who are high in dominance, self-confidence, a need to influence, who are able to articulate their goals and vision, perceived well, and have high expectations of their subordinates (House and Howell, 1992). Indeed, an extensive study investigating U.S. military personnel on exercise, conducted by Bass et al. (2003) found that platoon (a conventional unit of c. 30 soldiers) potency was positively correlated to TL. However, while these findings and methodologies are not being disputed, we question the uncritical and untested extension of this theory to SOF. TL implies followership and tacitly operates on the assumption that, in most situations, the leader of the group is expected to provide the answer to the tactical question posed. Notably, this form of leadership theory and the culture it engenders, runs counter to the ethos and organizational structure of SOF, because all members are encouraged to come forth with solutions to solving the mission, not just the leader. The SOF leader must be both adaptable and empowering because the operators they serve alongside need to trust their own initiative in hyper-dynamic environments (Zweibelson, 2017). In fact, each member of the SOF team will bring distinct leadership capability to the team-based execution that typifies special operations. Consequently, psychologists aspiring to work in this environment must have an appreciation of the group dynamics of SOF, like the SAS and elite conventional units, for example the pathfinders of the Parachute Regiment – then find theories that could potentially inform the design of a suitable intervention – if one is required.

### Special, not elite

For the sake of clarity, it is also important to distinguish between elite conventional forces (GPF) and SOF. Searle (2017) explain this in a straightforward way: an elite unit is very good at what they do, but they are not necessarily special. “Special,” implies different, rather than merely “better” (Searle, 2017, p. 11). Importantly, however, especially against the plethora of personal accounts in books and in the media in general, it is often very difficult for “outsiders” to understand the different levels at which conventional forces and SOF work. This is further compounded by differences in countries’ military cultures. Consequently, care must be taken when applying research formulated in starkly different environments, then applied to SOF without due consideration given to the unit’s level of expertise and the operational demands routinely placed upon them. Psychologists working in this operational sphere need to have the necessary cultural skill set to develop methods and approaches that can be conceptualized, monitored and justified against a very different set of performance challenges.

Of course, we also acknowledge the potential for confusion, especially to civilians unused to the nomenclature, status and “pecking order” of different units. Given that we have established SOF has an entirely different approach than conventional armed forces, due to their selection, mission sets and command structure, we would suggest another approach when working with SOF. Indeed, we would contend that you should not research or conduct applied work with conventional forces and then take the same approach and transfer it to SOF. It would be like doing applied work with soccer, for example how players are using their vision (scanning) in premier league (Jordet et al., 2020) and then trying to do identical interventions, based on those results with American football. There are superficial similarities (humans in a team sport for example) but, after that, the training we provide should be bespoke.

### Performance enhancement as wellbeing

This leads us to the second distinction in Part 1, namely the essential need for a focus on performance – not just mental health. Clearly a significant development has occurred, not only in sports but also in our society, regarding the way we treat mental health and welfare - and the military is no different (*cf.* Delima-Tokarz, 2016). Focusing on the mental health and welfare of military personnel is of course an important factor (Adler et al., 2011). Yet, it is no more or less important for SOF than it is for conventional forces. In contrast however, and against the increased risks inherent in their role, one of the most important “mental health” factors we might be overlooking is performance. Currently, according to our experience as practitioners and researchers working in this field, there is an imbalance in the number of mental health experts working with SOF, versus the number of *performance* psychologists. There are probably several reasons for this, historically and culturally, psychologists working for and in the military are primarily clinicians (Hacker Hughes et al., 2019). Anecdotally, our experience is that it is both easier and more encouraged for SOF operatives to consult a mental health clinician than it is to access psychology for performance enhancement.

This might be missing an important point. As important as fostering mental health is, we contend that the acquisition of a judiciously tailored performance psychology training for SOF is also an important prophylactic, necessary to engender mental health. For example, assuaging chronic worry and anxiety by using mental skills to plan more effectively enables operators to develop and refine their capacity to work in a pressured, expertise-led, high-performance environment. To summarize, it is our contention that SOF operatives require at least as much (if not more) *performance* psychology as clinical mental health support, especially when the performance approach is holistic (Kelly et al., 2013); meaning that the development of the whole person is imperative, not solely restricted to the operator’s performance domains by a technical focus (Miller and Kerr, 2002). In short, one’s mental health is likely to be bolstered by increased confidence that you will survive the mission!

### Part 2: Understanding the unique requirements on the performance arena for SOF

#### Selection as a basis for developing a tailor-made performance psychology program

We would argue that the SOF selection process is a developmental experience, not just a rite of passage. Therefore, we would suggest an epistemological chain of thinking, hopefully leading to coherent developmental activities (*cf.* Grecic and Collins, 2013), influenced by the experimental existential psychology paradigm (Koole et al., 2006). First and foremost, with the notion that humans construct their own reality based on the context, experiences and how they perceive their future. Our contention being that the SOF candidates learn and grow from the selection process (as well as achieving selection) and continue this process throughout their career as they gain experience and develop their expertise. Indeed, we would argue that selection could be more accurately described as an experiential learning process (Kolb, 2015), developing crucial individual *and* collective self-efficacy (Bandura et al., 1999). Candidates are required to find the wherewithal to continue when most of the training cadre has withdrawn; indeed, a majority are failed with the challenge to learn, return and meet the standard in the future. Therefore, our argument is that, in SOF especially and to some degree in elite conventional units’ selection processes, selection is not just about finding the right person for the job but also, and of equal importance, providing the opportunity for growth (or voluntary withdrawal) that the selection process and subsequent training presents. Furthermore, we would suggest interesting parallels with elite sports talent development (TD) (Toering et al., 2009; Larsen et al., 2012). Viewing the SOF candidates as *potentials*, where one of the most important process markers is the opportunity to develop your psycho-behavioral skills, for example, perspective, control and confidence during difficult circumstances (Savage et al., 2021). Reflecting this contention, one possibility of future research could be the organic development of specific psycho-behavioral characteristics of excellence (PCDEs) in a SOF context (*cf.* MacNamara et al., 2010). Examining the arena for development that arises through the SOF selection process itself, it is obvious these challenges offer opportunities for operators to develop (and even discover) their psycho-behavioral skills and gives opportunity for further development and refinement. This is especially applicable given the young age and even occasional dysfunctional upbringing which typifies many SOF candidates (e.g., McNab, 1995). Notably, however, use of this clear, skills-based approach, providing users with a flexible “hand of cards” with which to face myriad challenges, may be more appropriate than a causally attributed single construct focus such as resilience, mental toughness or hardiness (Collins et al., 2018a).

In relation to this emerging research, we would suggest an increased awareness around the relevance and applicability of mechanistic constructs, that seemingly carry face validity. For SOF and elite conventional units, understanding the context of increasing hardiness, resilience or mental toughness is important, either *a priori* or once the selection and training process is completed. We have a concern that these constructs have become almost ubiquitous in their application. As one example, resilience has been increasingly endorsed because of work carried out in the school of positive psychology (Seligman, 2019) and is predicated upon the ability to cope adaptively, having personal control, hardiness and available social support. The possession and the ability to employ allied strategies successfully is proposed to result in higher resilience, leading to decreased mental symptoms and to enable career and personal success (Reivich et al., 2011). Implicitly, the onus is placed on the individual to address the psychological consequences of their military deployments (Friedman and Robbins, 2012). Turning to recent specific military research shows that hardiness is claimed as another advantage in military leadership selection (Nordmo et al., 2022) and that mental toughness (MT) is an important factor when it comes to “behavioral perseverance in SOF selection” (Gucciardi et al., 2021, p. 165). It seems like researchers are keen to attribute their own particular construct to these performers.

The most substantive effort to deploy resilience in a military setting was undertaken with the introduction of the U.S. Army’s Global Assessment Tool (GATS). GATS was deployed to support the Comprehensive Soldier and Family Fitness program (CSF-2). Commencing in 2009, this 105-item questionnaire was distributed to one million soldiers, annually. Included in this initiative is online and didactic resilience training, with the latter being delivered by trained military personnel. Criticism regarding the content of GATS was acknowledged, but it was reported that the effect of GATS, and the sub-components relating to resilience, were unlikely to be fully understood for at least a decade (Lester et al., 2011). In another example, an evaluation of the Canadian Armed Forces’ Road to mental resilience program (R2MR; Fikretoglu et al., 2019), resulted in contraindicatory empirical evidence. It stated that the data presented “a very complex picture in which it is made evident that sensible, evidence-informed workplace mental health interventions such as R2MR may work under high fidelity conditions but may yield no discernible benefit or even inadvertent iatrogenic effects if implemented poorly or without sufficient consideration to the larger organizational context” (Fikretoglu et al., 2019, p. 12).

We would therefore argue that, in particular for the SOF community, the experiential process and consequent personal learning is key, developing the adaptability needed in their unique work environment (Ward et al., 2018) rather than the development of a specific construct (*cf.* the hand of cards idea presented earlier). In summary, certain specific skills needed to get through a selection and training pipeline might not be the best ones developing further in a long operational career. One example could perhaps be described through the idiom *to keep a stiff upper lip* implying an innate ability to push yourself through mental and physical hardship. Of course, this ability is crucial to pass SOF selection and very useful to tap into at times. On the other hand, holistic development of a broad range of skills is not only important for a long career, but also in a longevity perspective. There is no current research to support this notion in a military context, although parallel findings from potentially equally pressured selection environments are available. For example, researching the draft of quarter backs and studying the plethora of data from the last 40 years, shows no correlation between the draft pick based on a certain set of attributes and subsequent performance. This could indicate that what you select on might not be what is eventually needed for developing optimum performance (including health) over time (*cf.* Berri and Simmons, 2011). Furthermore, this contrasts with the *psychometric* approach to these challenges (*cf.* Collins and Cruickshank, 2017). That these states, or even traits can be measured in a meaningful but singular way that provides positive impact on the operators themselves. Thus, while the military promotion and training of resilience is clearly widespread, there has been criticism that there is an over-reliance on this as a single mode of measurement (Friedman and Robbins, 2012), meaning that soldiers and SOF operators’ experiences and reflections over the lifecycle of a deployment remain underexplored. Reflecting this and earlier criticisms, a recent study highlights that, while combat exposure is more common amongst SOF, there are similar or even more positive levels of mental health issues when contrasted to GPF (Dretsch et al., 2020).

So, based on the available literature, it seems like some academics are looking in and doing research on, not with or for the military (*cf.* Collins and Kamin, 2012). We need to be aware of what we are doing and how, especially on SOF, as we have highlighted earlier in the article (Searle, 2017). In fact, at least from our experience working with these populations, SOF operators do not require more mental toughness, resilience, or hardiness as essential precursors: they are not born Marvel Avengers! Rather, they need to balance the acquisition of expertise and the need to perform at a high level through a process of continuous growth.

#### Programming training in the SOF context

Another perhaps overlooked perspective is the mechanism underpinning the impacts of stress; historically Selye (1936) conceptualized managing stress as the maintenance of a balanced mental and emotional state. Interestingly, in an extension to this position Kobasa (1979) proposed that stress and emotion were linked to the concept of homeostasis. Consequently, applied practitioners, through talking therapy and selected homework tasks, sought to enable the maintenance of an equilibrium, in and for their clients. However, for individuals working in extreme environments, striving to reacquire homeostasis following periods of training and/or performance induced stress may be challenging or, perhaps, even counterproductive.

More recently, research on the concept of allostasis (McEwen et al., 2012; Sterling, 2012; Kleckner et al., 2017; Guidi et al., 2021) has suggested that the brain/body system adapts to change, rather than seeking a return to a homeostatic equilibrium. Thus, prolonged combat/performance experience is hypothesized to alter the set point, which is termed the allostatic load (Ursin and Eriksen, 2004; McEwen et al., 2012). This theory is emerging as a useful means of interpreting the potential psycho-physiological consequences of prolonged exposure to stress in frontline military populations (McEwen et al., 2012). To summarize, further research and a more thorough appreciation regarding stress response in this population is required. This implies understanding both prolonged combat stress and stress exposure in shorter, more intense experiences against the chronic “daily grind” stress. Consequently, an individualized and context-variable approach that investigates the antecedents and consequences of training and performance-induced stress is essential.

To prepare effectively, SOF need to imitate operational conditions, but they simply do not always know, based on their operational cycle when, where or what exactly their missions will look like until it is there. Therefore, a failure to understand SOF methods in training can seriously affect the desired outcome. Indeed, some SOF training takes place in sub-optimal settings; for example, drilling with live ammunition but in a controlled environment, is a well-established tool in the SOF training canon. Therefore, using methods and measurements that work very well on an individual Olympic level athlete preparing for a well-structured, understood and tightly scheduled challenge, will probably not have the same impact in a SOF training or operational context. Performing in the military “arena” is the opposite of performance in the Olympics, at least in terms of predictability. To summarize, the approach to the above-mentioned methods needs to be carefully tested and implemented for a training context, to maximize the effects of training, if the operators are in a controlled environment. Once the mission is given the green light, SOF (and elite) operators need to perform under immense pressure with a serious risk to their own life. In essence, it is very complex within its inherent simplicity: if you cannot perform at an excellent level during controlled training, you will not become a better operator during combat. We need to look at the specifics of the SOF population. A possible solution is to understand the interaction between the physical and the mental aspect and be able to think creatively regarding the different components of periodization (Collins et al., 2018b; Kiely, 2018) to ensure proper integration of performance psychology *for* SOF.

In summary, methods/methodologies are *not* directly transferable from conventional units or elite athletes. On the contrary, we would argue it is less disruptive and therefore better practice to conduct training as usual, than to integrate factors that have the potential to reduce the quality of training – because the context is misunderstood, even in the short-term.

### Part 3: Future considerations for developing performance psychology for SOF

There are of course SOF performance initiatives supported by NATO Governments currently underway that are official; for example, Canadian Special Forces (CANSOF) have developed the Special Forces Mental Agility program (SOMA) based on what they recognise as the unique requirements of SOF. This program is distinct from the well-established conventional Canadian Armed Forces program R2MR (Fikretoglu et al., 2019). However, SOMA is only a two-day group-based course, but perhaps a good start towards an integrated performance psychology package (Mattie et al., 2017, 2020).

In terms of other performance initiatives, in the United States, in 2009 a SOCOM initiative termed Tactical Human Optimization, Rapid Rehabilitation and Reconditioning (THOR3) program was launched. THOR3 was based on the primary assumption that: “Humans are more important than hardware” (Kelly et al., 2013). Initially, the main emphasis of this program was on physical development. More recent extensions to the THOR3 program widened its scope and have been outsourced to a civilian company (KBR, 2020). The program is now described as: “Under the United States Special Operations Command (USSOCOM), the Preservation of the Force and Family (POTFF) program.” This initiative intends to provide a holistic method to address the short and long-term well-being of these (SOF) operators (cit. KBR, 2020). KBR’s intention is to work in an interdisciplinary manner, with strength and conditioning coaches, dieticians, physiotherapists and psychologists. Based on available, but commercially sensitive information, the evolution of THOR3 can be viewed as a step in the right direction, at first glance.

In other relevant work, Greene (2019) highlights the role of performance coaching and details the specific needs that the SOF community have in terms of coached performance interventions. Importantly, however, many of these interventions have emerged from atheoretical or non-empirical foundations. In sum, it seems the available literature and research on different interventions for SOF need a higher degree of focus, especially given the high stakes environment they espouse to service.

### So, what sorts of things might we want to do?

For a start, we have got to make sure that what we are working on is in fact special, and the training we, as psychologists, are advocating is bespoke. It is important to develop a solid theoretical and empirical base to the work required: especially if performance psychology for SOF is to be developed to its full potential. Below are some suggestions as to how to proceed:

Advanced courses are often a part of the training pipeline both for SOF and conventional elite soldiers; however, it is important to evaluate the goodness-of-fit of mental skills training to determine its potential, and to identify any associated limitations to the dynamic, outcome-based training environment. There is substantial evidence that mental skills training is crucial for top performance in sports (Vealey, 2012; Hamilton et al., 2020) but its integration and application in military settings is, we would suggest, significantly under researched.

Yet, there is clear potential to develop individually tailored, bespoke, performance psychology protocols for integration into the SOF operational and training spheres. Goodwin (2008) presents an understanding of the difference in granularity when it comes to understand the military “arena” and we see this notion as very valuable when looking at SOF requirements as opposed to GPF. Another important factor to consider is the SOF training regimen. It is necessary to fully understand the requirements made of an SOF operator and their individual responses to training stimuli. Such individuals are required to function cognitively at a high level for hours at a time, in a high-stakes environment, but with less food and rest than sport-oriented high performers. Moreover, they are expected to perform whilst also coping with high levels of stress, both in training and in “competition.” Once again, bespoke evaluation and *much* more care in transferring methods from one domain to another is warranted.

One very interesting research study, relevant to SOF, that investigated the execution of complex tasks in a high-stakes, stressful environment investigated both High Altitude Low Opening (HALO), and High-Altitude High Opening (HAHO; Clemente-Suárez et al., 2017) parachute jumps. Both scenarios require specialized skillsets that call upon high levels of motor and cognitive skills. In this study, a panel of psychophysiological variables were assessed: the primary finding indicating that HAHO jumps result in a decrease in cortical arousal and a higher blood lactate concentration than HALO jumps. This infers that complex decision-making tasks are potentially more exacting when executing HAHO than HALO jumps. We offer no conclusions from this seemingly counterintuitive finding. This is, however, a rare but important example of research that can be used to inform changes to current training and operational practices, but more research of this type is required (Clemente-Suárez et al., 2017). Lastly, looking into the developing research on PCDEs (MacNamara et al., 2010) and conducting context specific research on SOF could inform about different methods of developing the talents selected for the SOF training pipeline.

### Understanding more about the operators’ cognitive demands in extremis

Learning about the subjective experience of veteran operators is crucial to developing and expanding performance psychology in this context. We need to develop an in-depth understanding, from the perspective of the SOF operator, as to their cognition in preparation for and performance in combat. Accessing this insight and experience is therefore a key resource. Tacit knowledge, defined by Grene (1971) as knowing more than you can tell, can be analyzed using established methodologies such as applied cognitive task analysis (ACTA – Militello and Hutton, 1998). Using a combination of interviews and graphics (if appropriate), the experienced operator can be guided to explore their tacit assumptions in training and following operations. In addition, the process can reveal the factors that inform decision-making, individually and collectively, in response to the mission parameters and objectives. Potentially, results such as these hold considerable value as to the design of an appropriate functional performance psychology training program, especially where such work shows possibly dysfunctional variations in working practice across experienced personnel (*cf.* Martindale and Collins, 2014).

Less formal, though still important, is the use of storytelling to create and share experiences among communities of practice (Li et al., 2009). While this informal method is easy and straightforward, a methodology still needs to be applied to record and share insights for training purposes. Therefore, tacit knowledge can be gleaned using such techniques and shared among experienced and neophyte operators to accelerate individual and collective expertise.

SOF is undoubtedly a special community, but it is currently not doing itself justice. Applied psychological research needs to be conducted in partnership with the SOF community –not through them, or on them (*cf.* Collins and Kamin, 2012).

### Longitudinal studies in a natural training cycle

A case in point is to determine if, how, when and, most crucially, why performance psychology can be used to potentially determine and alleviate the impact of the training and performance cycle that is fundamental to the SOF workplace. In short, how can human performance psychology be integrated into current working practices? From this, we would be able to suggest and evolve optimal impact. Just as with clients in occupational and clinical settings, careful case conceptualization and a clear understanding of the cognitive activity associated with high level performers will be essential to determine optimum support (*cf.* Martindale et al., 2017). Hence, there is a need for longitudinal studies that look at the preparation of SOF, prior to and following their deployment – to engage in thorough debriefs, potentially using specialized techniques developed by psychologists working in allied fields. Professionally, as we develop an informed workplace culture and praxis, we need to strive to do a first-rate job, informed and in possession of not just the “how,” but also the “why” SOF do it their way.

## Conclusion

SOF deserve their own conceptual subset within the domain of performance psychology; at least as much as dancers are researched separately to weightlifters! Importantly, such a careful and well considered application will also benefit elite conventional units because it can inform best practice(s) as research to enhance military performance is peer-reviewed (where possible) and good practice is shared more widely. We hope to have evidenced that existing research is inadequate. Where it has been carried out in relation to SOF operatives, it has seemingly not been carried out with the intention to enhance performance but rather (we would argue) to extend the scope of a psychological construct in conjunction with a clearly special population. As with other performance domains, it is not practical to simply export sport, and other sub-sets of psychology directly into the SOF domain (*cf.* Pecen et al., 2016). Indeed, relying on constructs which are confusing and lack empirical evidence in how to train for optimum impact, such as the global application of resilience, will almost inevitably miss the mark and potentially stifle more promising avenues of research. This quite apart from the potentially severe impacts (when compared to sport) on the operators in question. As stressed by Collins and Kamin (2012), if we are an applied field, we need to put performer’s needs first. Our approach needs to be combined with a solid epistemological chain that underpins the first SOF principle: humans are more important than hardware.

## Author contributions

All authors listed have made a substantial, direct, and intellectual contribution to the work and approved it for publication.

## Conflict of interest

The authors declare that the research was conducted in the absence of any commercial or financial relationships that could be construed as a potential conflict of interest.

## Publisher’s note

All claims expressed in this article are solely those of the authors and do not necessarily represent those of their affiliated organizations, or those of the publisher, the editors and the reviewers. Any product that may be evaluated in this article, or claim that may be made by its manufacturer, is not guaranteed or endorsed by the publisher.
